# Impact of Time of Surgery on the Outcome after Surgical Stabilization of Rib Fractures in Severely Injured Patients with Severe Chest Trauma—A Matched-Pairs Analysis of the German Trauma Registry

**DOI:** 10.3389/fsurg.2022.852097

**Published:** 2022-05-11

**Authors:** L. Becker, S. Schulz-Drost, C. Spering, A. Franke, M. Dudda, O. Kamp, R. Lefering, G. Matthes, D. Bieler, on behalf of the Committee on Emergency Medicine, Intensive Care and Trauma Management (Sektion NIS) of the German Trauma Society (DGU)

**Affiliations:** ^1^ Department of Trauma Surgery, Hand and Reconstructive Surgery, University Hospital Essen, Essen, Germany; ^2^ Department of Trauma Surgery, Helios Hospital Schwerin, Schwerin, Germany; ^3^ Department of Trauma and Orthopedic Surgery, University Hospital Erlangen, Erlangen, Germany; ^4^ Department of Trauma Surgery, Orthopaedics and Plastic Surgery, University Hospital Göttingen Medical Center, Göttingen, Germany; ^5^ Department of Trauma Surgery and Orthopaedics, Reconstructive and Hand Surgery, Burn Medicine, German Armed Forces Central Hospital Koblenz, Koblenz, Germany; ^6^ Institute for Research in Operative Medicine (IFOM), Witten/Herdecke University, Cologne, Germany; ^7^ Department of Trauma Surgery and Reconstructive Surgery, Ernst von Bergmann Hospital, Potsdam, Germany; ^8^ Department of Orthopaedics and Trauma Surgery, Heinrich Heine University Hospital, Düsseldorf, Germany

**Keywords:** rib stabilization, chest trauma, rib fracture, multiple trauma, SSRF, thoracic trauma

## Abstract

**Purpose:**

In severely injured patients with multiple rib fractures, the beneficial effect of surgical stabilization is still unknown. The existing literature shows divergent results, and the indication and especially the right timing of an operation are the subject of a broad discussion. The aim of this study was to determine the influence of the time point of surgical stabilization of rib fractures (SSRF) on the outcome in a multicenter database with special regard to the duration of ventilation, intensive care, and overall hospital stay.

**Methods:**

Data from the TraumaRegister DGU collected between 2010 and 2019 were used to evaluate patients above 16 years of age with severe rib fractures [Abbreviated Injury Score (AIS)  ≥ 3] who received an SSRF in a matched-pairs analysis. In this matched-pairs analysis, we compared the effects of an early SSRF within 48 h after initial trauma vs. late SSRF 3–10 days after trauma.

**Results:**

After the selection process, we were able to find 142 matched pairs for further evaluation. Early SSRF was associated with a significantly shorter length of stay in the intensive care unit (16.2 days vs. 12.7 days, *p* = 0.020), and the overall hospital stay (28.5 days vs. 23.4 days, *p* = 0.005) was significantly longer in the group with late SSRF. Concerning the days on mechanical ventilation, we were able to demonstrate a trend for an approximately 1.5 day shorter ventilation time for patients after early SSRF, although this difference was not statistically significant (*p* = 0.226).

**Conclusions:**

We were able to determine the significant beneficial effects of early SSRF resulting in a shorter intensive care unit stay and a shorter length of stay in hospital and additionally a trend to a shorter time on mechanical ventilation.

## Introduction

In the last few years, surgical stabilization of rib fractures (SSRF) is increasingly coming into the focus of scientific research from multiple perspectives. Researchers from different medical disciplines such as orthopedic surgery, trauma surgery, and thoracic or cardiac surgery are using a multidisciplinary approach to find the best treatment for rib injuries. Since the beneficial effects of SSRF in general are accepted in certain patient populations by most researchers, the main questions that are still hot topics of discussion are the right indications and the right time to perform SSRF (1, 2). For example, de Jong et al. demonstrated in a systematic review that, besides a precise indication for SSRF, an early time point of surgical therapy for multiple rib fractures is decisive in whether the patient will benefit or not (3). In a multicenter study in 2018, a daily increase of pneumonia and risk for long-term ventilation was shown for patients with a flail chest who received no or delayed surgical treatment, so the timing of an SSRF seems to be more important than previously thought or appreciated in clinical practice (4). On the other hand, the critical review of the evidence for the treatment of serial rib fractures by Beks et al. stated that a general surgical treatment for patients with ≥3 rib fractures showed no statistically significant advantage independent of the time of surgery (5). In general, the level of evidence in the literature is rather low so far, as most of the existing studies lack sufficient sample sizes or compare operative vs. nonoperative treatment independent from the time of surgery (6).

Data from our group showed in an analysis of the TraumaRegister DGU that the current clinical practice for the treatment of most of these patients was not according to the recent literature and showed a delay in the time for operative care of well over 48 h. This may lead to an increased rate of complications and a longer stay in the intensive care unit (ICU) and the hospital in general (7).

In the light of the heterogeneous study situation concerning the time point of SSRF as well as small case numbers in most of the existing literature works, the aim of this analysis is to evaluate the influence of the time of surgery in patients with multiple rib fractures by conducting a matched-pairs analysis in the TraumaRegister DGU.

## Materials and Methods

The TraumaRegister DGU of the German Trauma Society (Deutsche Gesellschaft für Unfallchirurgie, DGU) was founded in 1993. The aim of this multicenter database is pseudonymized and standardized documentation of severely injured patients.

Data are collected prospectively in four consecutive time phases from the site of the accident until discharge from the hospital: (A) prehospital phase, (B) emergency room and initial surgery, (C) intensive care unit, and (D) discharge. The documentation includes detailed information on demographics, injury patterns, comorbidities, pre- and in-hospital management, the course on intensive care unit, and relevant laboratory findings including data on transfusion and outcome of each individual. The inclusion criterion is admission to the hospital via an emergency room with subsequent ICU/IMC care or arrival at the hospital with vital signs and death before admission to an ICU.

The infrastructure for documentation, data management, and data analysis is provided by AUC—Academy for Trauma Surgery (AUC—Akademie der Unfallchirurgie GmbH), a company affiliated with the German Trauma Society. The scientific leadership is provided by the Committee on Emergency Medicine, Intensive Care and Trauma Management (Sektion NIS) of the German Trauma Society. The participating hospitals submit their data pseudonymized into a central database via a web-based application. Scientific data analysis is approved according to a peer-review procedure laid down in the publication guideline of the TraumaRegister DGU.

The participating hospitals are primarily located in Germany (90%), but a rising number of hospitals in other countries contribute data as well (at the moment from Austria, Belgium, China, Finland, Luxembourg, Slovenia, Switzerland, The Netherlands, and the United Arab Emirates). Currently, more than 35,000 cases from nearly 700 hospitals are entered into the database per year.

Participation in the TraumaRegister DGU is voluntary. For hospitals associated with TraumaNetzwerk DGU, however, the entry of at least a basic data set is obligatory for reasons of quality assurance.

We selected patients aged 16 years and older with rib fractures (AIS ≥3; coding ≥3 fractured ribs or unilateral/bilateral flail chest) from Germany, Switzerland, and Austria who were treated between 2010 and 2019. Only patients from hospitals that used the standard data set for documentation were included since the reduced basic data set does not contain any information on operative care. Patients with no or a minor thoracic trauma (AIS 0–2; that is, a maximum of 1–2 fractured ribs) were excluded. Children under 16 years of age or undocumented age were also excluded, as were patients who were transferred out to another hospital early after the initial trauma (<48 h) or transferred to the treating hospital later than 5 days after trauma. Additionally, all patients with an SSRF later than 10 days after trauma and patients who died within the first 10 days were excluded to eliminate confounding events.

To obtain a better comparison of the time point of SSRF, a matched-pairs analysis was carried out to sharpen the statement of any differences and to increase comparability. To obtain groups that were as comparable as possible, the surgically stabilized patients were paired in groups with a patient who received SSRF within the first 48 h vs. 3–10 days after trauma with regard to the following criteria:

• age group (16–54, 55–69, and older than 69 years),
• severity of the rib fractures (AIS 3/4/5),
• injury severity (AIS) in four body regions (head, thorax, abdomen, and extremities), and
• RISC2 (0–4/5–10/11–15/16–20/21–49, and over 49).

To take the different influences of the injury patterns and their severity into account, pairs were matched using the RISC2 score. Each head, abdominal, and extremity injury was assigned a counterpart depending on its severity. The matching categories, with regard to the severity of the injury, were defined with AIS 0–2, 3, 4, and 5, respectively. The procedure for rib injuries was analogous, with only AIS codes 3, 4, or 5 being used here, since minor rib fractures (AIS 0–2) were excluded.

The AIS was introduced in the 1960s to describe the severity of an injury based on an anatomical scoring system coding type, location, and the severity of an injury. Besides the anatomical region and the type of injury, the severity is scaled from one to six, one being a minor injury and six being maximal (8). Based on the AIS, the Injury Severity Score (ISS) can be calculated to assess the severity of multiple injuries, to estimate the morbidity and mortality, or to define whether a patient is polytraumatized. Therefore, the three most severely injured body regions have their score (AIS) squared and added together to produce the ISS, ranging from 1 to 75. Major trauma is defined as an ISS ≥15 (9, 10).

In 2014, the Revised Injury Severity Score, version II (RISC II), was introduced by Lefering et al. as a model for the prediction of the risk of death in severely injured patients. It consisted of 15 different prognostic factors and was able to demonstrate a superior quality of predicting the risk of death compared to more traditional scoring systems, e.g., ISS, RISC, or Trauma Injury Severity Score (TRISS) (11).

### Statistics

Primary endpoints were the length of hospital stay, ICU treatment, and the duration of ventilation. Secondary endpoints were general data of the patient collective, trauma mechanism, blood transfusions, and death >day 10 after trauma.

The statistical evaluation was carried out with SPSS (Version 23, IBM Inc., Armonk, NY, USA). The data of the matched patients were compared with the aid of the paired samples Wilcoxon test. The level of significance was set at 5% (*p* < 0.05). Missing values were not replaced but excluded on a case-by-case basis. This study follows the current publication guidelines of the TraumaRegister DGU and is registered under the TraumaRegister DGU project ID 2021-007.

## Results

**Figure 1:**
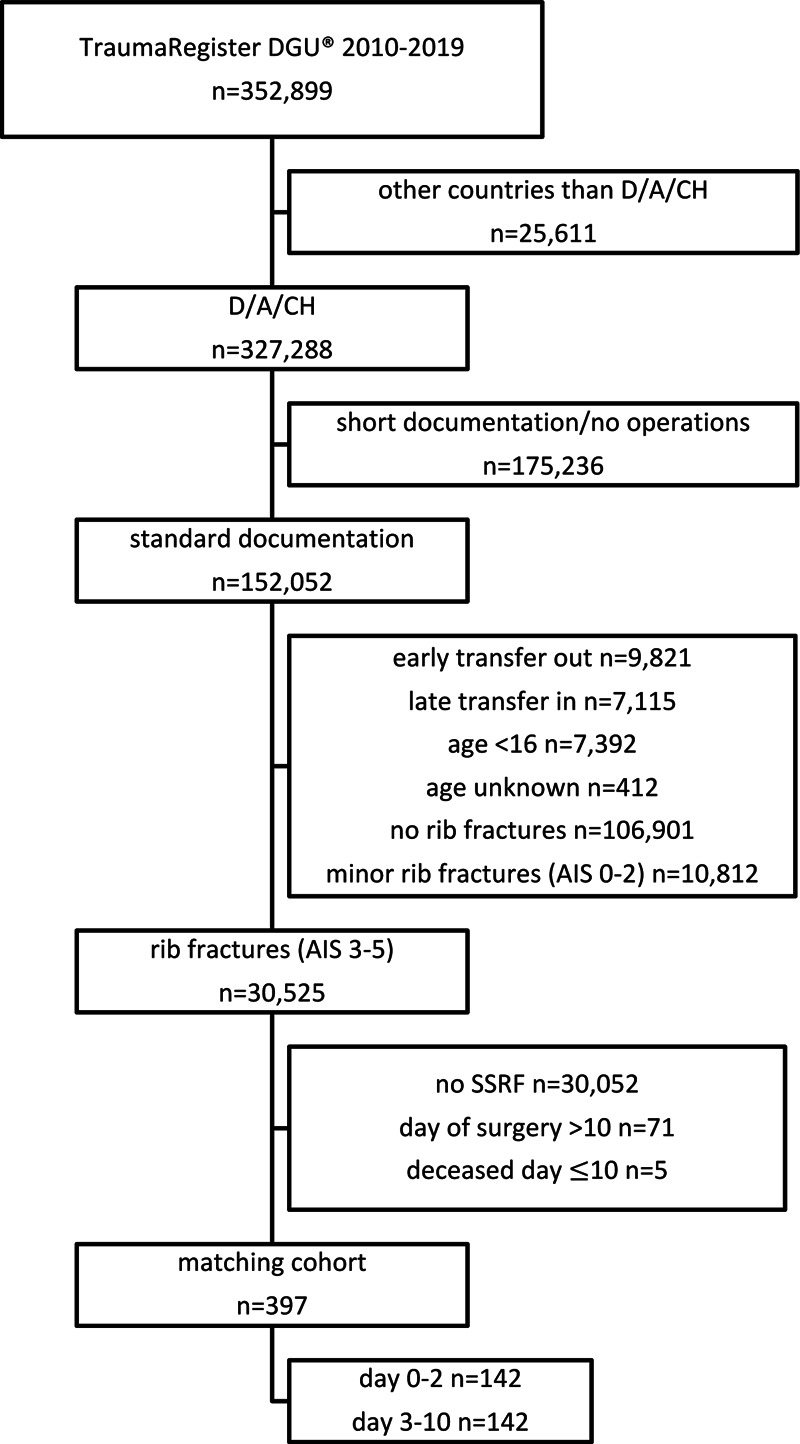
Selection process of the patient collective before matching. D, Germany; A, Austria; CH, Switzerland; AIS, Abbreviated Injury Scale; SSRF, Surgical Stabilization of Rib Fractures.

The complete data set of the TraumaRegister DGU for the period 2010–2019 consists of 352,899 patients. First, we excluded all patients treated outside Germany, Austria, or Switzerland (*n* = 26,611) to eliminate the influence of different medical systems. In the next step, the limited data sets of the short documentation and all patients without any operative treatment were identified (*n* = 175,236). All patients with an early transfer (<48 h) out (*n* = 9,821) of or late transfer (>5 days after trauma) in (*n* = 7,115) the treating hospital were excluded. Children with age <16 years (*n* = 7,392) or patients with unknown age (*n* = 412) were eliminated from the data pool as well. Additionally, patients with no (*n* = 106,901) or minor rib fractures (AIS 1–2, *n* = 10,812) and consecutively all patients with severe rib fractures (AIS ≥ 3) but without SSRF (*n* = 30,052) were excluded. In the final step, we removed all data sets with an SSRF later than 10 days after the initial trauma (*n* = 71) or patients who died on day 10 or earlier (*n* = 5). After applying all inclusion and exclusion criteria, 397 patients were left for matching. We were able to find 142 matched pairs using the above-stated matching criteria regarding age, AIS of the rib fractures, injury severity in four body regions, and the RISC2 (**Figure 1**).

The mean age of the matched pairs was 57.5 years; approximately, 80% were male and 20% were female. Concerning the severity of the injuries, the mean ISS was 26.3 and the calculated risk of death based on RISC II was 9.3% in the matched-pairs cohort. Regarding the mechanism of injury, approximately half of the included patients suffered their injuries in a road traffic accident (RTA). During the initial resuscitation in the trauma room on day 0, only 12.3% received a transfusion of red blood cells (RBCs). Eight patients or 2.8% of the matched-pairs cohort of a total of 284 patients deceased in the further course of treatment after day 10. The distribution of AIS in the different anatomical regions is shown in **Table 1**. No significant differences could be found for the data outside the matching criteria.

**Table 1:** Distribution of AIS.

	SSRF days 0–2	SSRF days 3–10	Total
AIS thoracic 3	*n* = 44 (31.0%)	*n* = 44 (31.0%)	*n* = 88 (31.0%)
AIS thoracic 4	*n* = 63 (44.0%)	*n* = 63 (44.0%)	*n* = 126 (44.0%)
AIS thoracic 5	*n* = 35 (24.6%)	*n* = 35 (24.6%)	*n* = 70 (24.6%)
AIS head ≥3	*n* = 21 (14.8%)	*n* = 28 (19.7%)	*n* = 49 (17.3%)
AIS abdomen ≥3	*n* = 14 (9.9%)	*n* = 15 (10.6%)	*n* = 29 (10.2%)
AIS extremities ≥3	*n* = 31 (21.8%)	*n* = 17 (19.0%)	*n* = 58 (20.4%)

*AIS, Abbreviated Injury Scale.*

The length of stay in the intensive care unit (mean 16.2 days vs. 12.7 days, *p* = 0.020) and the overall hospital stay (mean 28.5 days vs. 23.4 days, *p* = 0.005) were significantly longer in the group with SSRF on day 3 or later. Concerning the days on ventilation, no statistically significant difference between the two groups was measured. On the other hand, we were able to demonstrate a nonsignificant trend (*p* = 0.226) for an average of 1.5 day shorter ventilation time for patients after early SSRF (**Table 2**).

**Table 2:** Matched-pairs groups: surgical stabilization on days 0–2 vs. 3–10.

	SSRF days 0–2	SSRF days 3–10	Total	
Age in years	57.8 (SD 15.3)	57.3 (SD 14.5)	57.5 (SD 14.9)	
RISC2	8.97 (SD 18.19)	9.72 (SD 15.69)	9.34 (SD 16.96)	
ISS	26.17 (SD 10.21)	26.42 (SD 10.92)	26.3 (SD 10.55)	
Ventilation days	7.49 (SD 10.07)	8.99 (SD 11.62)	8.24 (SD 10.88)	*p* = 0.226
ICU days	12.70 (SD 12.46)	16.23 (SD 15,.72)	14.46 (SD 14.27)	*p* = 0.020*
LOS hospital days	23.44 (SD 15.23)	28.50 (SD 18.45)	25.97 (SD 17.08)	*p* = 0.005*
Female	*n* = 28 (19.7%)	*n* = 30 (21.1%)	*n* = 58 (20.4%)	
Male	*n* = 114 (80.3%)	*n* = 112 (78.9%)	*n* = 226 (79.6%)	
RTA as MOI	*n* = 78 (55.3%)	*n* = 74 (53.2%)	*n* = 152 (54.3%)	
RBC transfusion on day 0	*n* = 21 (14.8%)	*n* = 14 (9.9%)	*n* = 35 (12.3%)	
Deceased > day 10	*n* = 3 (2.1%)	*n* = 5 (3.5%)	*n* = 8 (2.8%)	

* = significant. SSRF, Surgical Stabilization of Rib Fractures; RISC2, Revised Injury Severity Classification; ISS, Injury Severity Score; ICU, intensive care unit; LOS, length of stay; RTA, road traffic accident; MOI, mechanism of injury; RBC, red blood cells; SD, standard deviation.

There was no difference in the length of stay in the ICU or the hospital after the SSRF between early and late operative stabilization (mean ICU stay: 11.8 days vs. 11.7 days, *p* = 0.26, mean hospital stay: 22.4 vs. 22.9 d, *p* = 0.89), implying a delay of surgery leading to a longer stay in the ICU or hospital.

The distribution of the time of surgery of SSRF for the included patients on days 0–10 after the initial trauma is shown in **Table 3**.

**Table 3:** Distribution of SSRF days 0–10.

	SSRF days 0–2	SSRF days 3–10	Total
SSRF day 0	*n* = 54 (38.0%)	0 (0%)	*n* = 54 (19.0%)
SSRF day 1	*n* = 32 (22.5%)	0 (0%)	*n* = 32 (11.3%)
SSRF day 2	*n* = 56 (39.4%)	0 (0%)	*n* = 56 (19.7%)
SSRF day 3	0 (0%)	*n* = 28 (19.7%)	*n* = 28 (9.9%)
SSRF day 4	0 (0%)	*n* = 24 (16.9%)	*n* = 24 (8.5%)
SSRF day 5	0 (0%)	*n* = 20 (14.1%)	*n* = 20 (7.0%)
SSRF day 6	0 (0%)	*n* = 22 (15.5%)	*n* = 22 (7.7%)
SSRF day 7	0 (0%)	*n* = 21 (14.8%)	*n* = 21 (7.4%)
SSRF day 8	0 (0%)	*n* = 14 (9.8%)	*n* = 14 (4.9%)
SSRF day 9	0 (0%)	*n* = 6 (4.2%)	*n* = 6 (2.1%)
SSRF day 10	0 (0%)	*n* = 7 (4.9%)	*n* = 7 (2.5%)

*SSRF, Surgical Stabilization of Rib Fractures.*

## Discussion

The routine treatment for stable, nondisplaced rib fractures in the current clinical practice is conservative. This is covered by a broad consensus on this strategy in the literature. On the other side, the optimal treatment of chest wall injuries with multiple rib fractures and the advantages or disadvantages of its surgical treatment are a subject of broad discussion in the literature in the last years. Until today, only one international consensus statement on this topic is available, and the lack of evidence has led to no national or international guidelines existing so far. To make matters more complicated, a comparison of the recommendations and study results in the literature is elusive by very inconsistent treatment strategies (12–17).

Operative treatment of displaced rib fractures by means of SSRF has been known and used for a long time to reconstruct the chest wall and restore adequate respiratory mechanics in combination with a significant reduction of pain at the same time (18). Recent publications provided evidence of a positive effect on mortality and a better outcome for patients with SSRF compared to a nonoperative treatment (19–22). For example, DeFreest et al. showed in their study, also carried out as a matched-pairs analysis, a lower mortality of 2.4% vs. 11.1% for surgical treatment (18). Additionally, Beks et al. and Liu and Xiong demonstrated in their meta-analysis a significantly lower mortality rate for patients with SSRF. Beks stated the determined risk ratio of mortality as 0.41, and the odds ratio for mortality in the analysis by Liu was 0.28 (5, 23).

Contrary to these results, the systematic review of existing review articles by Ingoe et al. and a Cochrane analysis by Cataneo et al. were not able to find any advantage concerning the survival of patients receiving a surgical stabilization of multiple rib fractures. Both authors criticized the low level of evidence in the existing literature as a limiting factor for a clear statement favoring one of the two treatment strategies (6, 24).

Essentially all of the available publications regarding the surgical treatment of rib fractures are based on a patient population from controlled studies. This simulated framework and a patient selection bias could result in difficulties in detecting the beneficial effects of SSRF. We tried to eliminate these problems by using a matched-pairs analysis in a large, multicentered, and unselected population-based cohort.

Concerning the right time point for performing SSRF, Pieracci et al. showed a daily increasing risk of approximately 30% for pneumonia, 27% for long-term ventilation, and 26% for tracheotomy in patients with multiple rib fractures and a conservative or delayed operative treatment (4). The advantage of operative treatment is, accordingly to these findings, mostly described within the first days or weeks after the initial trauma. However, still, there is no proven evidence of a benefit in the long-term outcome compared to conservative treatment or late SSRF in the literature (6, 25, 26). In the latest publication of our group, it was shown that only a small portion of the patients with SSRF was operated on in the current clinical practice based on the data in the TraumaRegister DGU within the recommended 48 h after trauma (7). In general, there is only a limited number of studies focusing on the time point of surgery; most are comparing only an operative vs. a nonoperative treatment without a differentiation of the time of surgery. This issue is, for instance, covered in the publications of Iqbal et al., who demonstrated beneficial effects of SSRF within 48 h vs. later treatment, resulting in a shorter ICU stay, a lower rate of pneumonia, a reduced duration of ventilation, and a shorter hospital length of stay (27). Additionally, Majak and Naess stated that SSRF within the first 72 h might lead to a better outcome (28). The Japanese group around Otaka was able to demonstrate an association of a better in-hospital outcome for early surgical fixations, whereas later surgical fixation was not (29, 30). The first review article discussing early vs. late SSRF was published by Radomski and Pieracci in 2019, stating that an early SSRF within 72 h should be achieved but was only based on three publications at this point of time (31). Prins et al. as well concentrated their review on the time point of SSRF and were able to recommend early SSRF within 48 or 72 h the latest based on the total number of nine publications concerning this matter (32). In our matched-pairs analysis, we were able to confirm these results by determining the significant beneficial effects of early SSRF, resulting in a shorter ICU stay and a shorter length of stay (LOS) in the hospital and additionally a trend to a shorter time on mechanical ventilation.

## Limitations of the Study

Studies using the data set of the TraumaRegister DGU have multiple methodological limitations from the outset. All retrospective studies have only a limited potential for conclusions drawn from their data set. Especially, like the TraumaRegister DGU, if they are not specifically designed for the scientific question, here the extent of thoracic injuries. The morphology, anatomical localization, and the type of fracture are unknown since only the number of broken ribs and the presence or absence of other thoracic injuries like pneumothorax, haemothorax, or a flail chest are documented. Since the morphology of the rib fractures, especially the amount of dislocation, is crucial for indicating SSRF, this is one of the major limitations of this study. Additionally, only the ventilation days and no specific ventilation parameters are recorded in the data set of the TraumaRegister DGU. From the studied data set, it is impossible to clear the indication for the performed SSRF and whether it was based on radiological diagnostics of the fracture pattern and/or on functional parameters. Additionally, this registry contains no specific information about the technique used for SSRF.

Further studies, ideally a prospective randomized study, with a data pool specifically designed for thoracic trauma will be necessary to further investigate these questions.

## Conclusion

We believe that it is important to further investigate the impact of early vs. late operative treatment in patients with multiple rib fractures and other severe injuries of the chest wall. Even though the evidence is limited, it is becoming clearer that SSRF (given the indication) should be performed rather early than late. Patients who are treated according to the recommended indications and the time of surgery stated in the literature in the last years seem to benefit from this therapy regimen. In contrast to this, it can be assumed that patients with a late SSRF are those with a difficult course in their treatment and/or prolonged weaning, so the indication for this group must be questioned. These patients are more likely to show a rather poor outcome overall, and therefore, no difference can be demonstrated. Additionally, it must be expected that a late operation will result in a “second hit” for the patient who will subsequently have to remain in the intensive care unit for a longer period of time before he/she finally recovers.

## Data Availability

The data analyzed in this study are subject to the following licenses/restrictions: The TraumaRegister DGU of the German Trauma Society (Deutsche Gesellschaft für Unfallchirurgie, DGU) founded in 1993. The aim of this multicenter database is pseudonymized and standardized documentation of severely injured patients. The application for evaluation must be made in writing and will first be checked by the Arbeitskreis TraumaRegister for its fundamental feasibility. Subsequently, two reviewers from the Review Board of TraumaRegister DGU subject it to a careful internal peer-review process. Requests to access these data sets should be directed to traumaregister@auc-online.de.
